# Muscle development, regeneration and laminopathies: how lamins or lamina-associated proteins can contribute to muscle development, regeneration and disease

**DOI:** 10.1007/s00018-012-1190-3

**Published:** 2012-11-10

**Authors:** Magda Dubinska-Magiera, Magdalena Zaremba-Czogalla, Ryszard Rzepecki

**Affiliations:** 1 Laboratory of Nuclear Proteins, Faculty of Biotechnology, University of Wroclaw, 63/77 Przybyszewskiego Street, 51-148 Wroclaw, Poland; 2 Department of Animal Developmental Biology, University of Wroclaw, 21 Sienkiewicza Street, 50-335 Wroclaw, Poland

**Keywords:** Laminopathies, Muscle differentiation, Muscle regeneration, Muscle degeneration, Nuclear envelope, Lamin A, Emerin

## Abstract

The aim of this review article is to evaluate the current knowledge on associations between muscle formation and regeneration and components of the nuclear lamina. Lamins and their partners have become particularly intriguing objects of scientific interest since it has been observed that mutations in genes coding for these proteins lead to a wide range of diseases called laminopathies. For over the last 10 years, various laboratories worldwide have tried to explain the pathogenesis of these rare disorders. Analyses of the distinct aspects of laminopathies resulted in formulation of different hypotheses regarding the mechanisms of the development of these diseases. In the light of recent discoveries, A-type lamins—the main building blocks of the nuclear lamina—together with other key elements, such as emerin, LAP2α and nesprins, seem to be of great importance in the modulation of various signaling pathways responsible for cellular differentiation and proliferation.

## Introduction

Muscle degenerative disorders, characterized by the progressive loss of muscle strength and integrity, affect a significant part of the human population. The genetic basis of these diseases is very diverse. Muscular dystrophies are caused by the mutations in different genes encoding a wide variety of proteins such as extracellular matrix proteins, transmembrane and membrane-associated proteins, nuclear proteins and cytoplasmatic enzymes [108]. In this article, we focused on the muscular dystrophies which belong to the laminopathies and are connected with the nuclear proteins. The term laminopathies defines a group of diseases associated with mutations in the genes coding for lamins, proteins associated with their post-translational processing, or proteins interacting with the lamins [249, 250], also refer: [255]. As indicated by several studies, such mutations may affect development of tissues of mesodermal origin, mostly muscles and its regeneration [142]. In the light of these discoveries, it is essential for us to analyze current data and try to understand the underlying molecular mechanisms responsible for the involvement of lamins and their partners in myogenic processes. We focused on molecular mechanisms and pathways which are or may be affected by mutations in genes coding for nuclear envelope proteins.

## Lamins

The cell nucleus is separated from the cytoplasm by the nuclear envelope (NE), composed of the nuclear lamina (NL), an outer and inner nuclear membrane (ONM and INM, respectively), connected in the region of nuclear pore complexes (NPC). The NL is composed of lamins, which are type V intermediate filament proteins and are grouped into A- and B-types based on their biochemical properties, post-translational modifications and behavior during mitosis. Lamins are present in the nuclear interior as well [30]. They are expressed in all studied metazoans but are absent from yeast and plants. Three genes encode lamins in humans: *LMNA* for A-type lamins (lamin A, C and other isoforms like lamin C2, lamin AΔ10) [131], *LMNB1* for lamin B1, and *LMNB2* for lamin B2 [99, 131]. It is commonly known that in contrast to B-type lamins, which are ubiquitously produced in all cell types during embryonic and adult life, A-type lamins are highly expressed in differentiated tissues, particularly in skeletal muscle and in some adult stem cells, including mesenchymal stem cells, hair stem cells and satellite cells. A-type lamins are absent in other types of stem cells, including embryonic stem cells [205]. A-type lamins are not essential for cell proliferation. They play a crucial role in the exit from the cell cycle [75]. Apart from expression patterns, lamins differ in terms of the presence of a farnesyl tail. Lamins A and B terminate with a CaaX motif (where C is a cysteine, a is an aliphatic amino acid, and X is often hydrophobic residues). The maturation of lamins requires sequential enzymatic modifications of this motif: farnesylation, proteolytic cleavage and carboxy-methylation. B-type lamins retain farnesylation, whereas in the case of lamin A (lamin C does not have a farnesylation motif) [219] the farnesyl tail is clipped off by zinc metalloproteinase ZMPSTE24 [17]. The structure of all types of lamins is very similar and related to their cytoplasmic intermediate filament homologues: a conserved central α-helical rod domain containing four segments—1A, 1B, 2A and 2B—is flanked by two variable globular domains (the N-terminal head domain and the C-terminal tail domain) [2].

Most scientists agree that one of the main functions of lamins are to provide structural support to the nucleus, maintenance of nuclear shape and spacing of NPC. Over the years, numerous reports have suggested that lamins also take part in other process: chromatin organization, DNA replication, epigenetics, transcription, cell cycle regulation, cell development and differentiation, nuclear migration, and apoptosis [193]. Lamins together with integral membrane proteins of NE and associated proteins participate in the regulation of chromatin organization [220] and formation of chromatin microdomains associated with NE [49, 141]. Although the necessity of B-type lamins in certain cell functions has been recently challenged [111, 253].

Lamins do not play all of those functions alone but by interaction with many other proteins of NE and nuclear interior as well. Major group of proteins interacting with lamins belong to LEM domain proteins: LAP2 proteins, emerin and MAN1 (LEMD3), LEM2/NET25 protein. Lamins also interact with LAP1 proteins, LBR, SUN proteins, pRb, MLIP, NET39, actin, cyclin D3, cFos, Oct-1, SREBP1, MOK2, ING1, PKCα, JIL-1 and BicaudalD protein. Lamins may also possibly interact with predicted membrane proteins: LEM3, LEM4, and LEM5. Lamins can bind nucleic acids, chromatin and histones in vitro and DNA and chromatin in vivo (see [209, 256]. Lamins and protein complexes containing lamins can affect transcription through different mechanisms [261].

## Laminopathies: the involvement of lamins and lamina-associated proteins in development, maintenance and regeneration of muscle tissue

Laminopathies typically not only affect one tissues in isolated fashion, or several tissues in a generalized way (premature ageing syndromes—systemic laminopathies), but have also overlapping phenotypes. Based on the affected tissue, laminopathies can be classified into several categories: lipodystrophies, neuropathies, dermopathies, cardiomyopathies and muscular dystrophies [137, 248, 255]. The most common group is laminopathies of the muscular dystrophy type, which is the focus of this study. The first reports on laminopathies appeared in the 1990s and described the X-linked recessive form of Emery–Dreifuss muscular dystrophy (EDMD1, XL-EDMD; OMIM 31300). Since then, a growing number of research papers on related diseases have appeared, some of them affect solely muscle tissues. Following are some of such diseases: autosomal dominant form of EDMD (EDMD2, AD-EDMD; OMIM 181350) [23] autosomal recessive EDMD (EDMD3, AR-EDMD; OMIM 604929), cardiomyopathy dilated 1A (CMD1A; OMIM 115200) [31], limb-girdle muscular dystrophy type 1B (LGMD1B; OMIM 159001) [165] congenital-type muscular dystrophy (OMIM 613205) [158] and “heart-hand” syndrome (HHS; OMIM 610140) [198] All mentioned diseases are caused by the mutations in *LMNA* gene, and have different clinical phenotypes. EDMD is characterized by the clinical triad of slow progressive muscle wasting, cardiac conduction defects, and contractures at the elbows, ankles and neck [79]. LGMD1B appears during the first 20 years of life and is characterized by weakening of the shoulder and pelvic girdle musculature, age-related atrioventricular cardiac conduction disturbances, and dilated cardiomyopathy [165]. Dilated cardiomyopathy is a disorder which causes atrial fibrillation and cardiac arrest. Heart-hand syndrome is characterized by the association of congenital cardiac disease and limb deformities [203]. Moreover, the literature also describes heterogeneous clinical cases suggesting an overlapping continuum with different types of laminopathies. For example, patients with clinical features of lipodystrophy also exhibit cardiac and skeletal muscular alterations [236], one report describes a patient with features of myopathy and peripheral neuropathy [18].

It is challenging to explain the mechanisms by which mutant lamins contribute to the tissue-specific pathology, while A-type lamins are expressed in all or many differentiated somatic cell types. According to recent knowledge, three hypotheses have been postulated to explain how mutations in the *LMNA* gene cause tissue-specific diseases for example muscular dystrophies.

The so-called structural hypothesis assumes that mutations in A-type lamins alter NL integrity, leading to its structural weakness, which finally results in decrease in the ability of nucleus to resist high mechanical stress within cells, particularly within skeletal muscles [32, 122, 223].

The gene-expression model hypothesis assumes that, mutations in the *LMNA* gene alter regulation of gene expression during differentiation of tissues of mesodermal origin, especially muscles. This may also cause the disruption of the balance between muscle degeneration and renewal [53, 75]. The mechanism by which gene expression is regulated by the NE is based on direct interactions of A-type lamins and associated proteins with different transcription factors and chromatin modifying complexes. [7]. A-type lamins play an important role in the muscle by influencing the expression of genes necessary for muscle cells differentiation. Several studies undertaken over the past few years support the gene-expression model, including the investigations of associations of A-type lamins and their partners (e.g. LAP2α) with transcription factors, such as Rb protein [144]. Changes in mRNA expression were observed for patients with laminopathy [57]. Myoblasts with reduced lamin A/C or emerin are characterized by reduced levels of proteins important for muscle cells: MyoD, desmin, pRb, and M-cadherin [75]. Recently, it has also been shown that microRNA (short RNA molecules that regulate gene expression by binding to target mRNAs) transcriptome is affected in laminopathy patients [224]. MicroRNAs play a crucial role in cardiac and skeletal muscle function. Some miRNAs are highly expressed in striated muscles, where they control the proliferation and differentiation balance [46]. Moreover, it is known that nucleoplasmic lamins colocalize with RNA splicing factor [120].

Some authors suggest a third, “combined theory” which connects both hypotheses [75, 145]. There are also other hypothesis that propose that lamins regulate tissue homeostasis, and lamin mutants impair adult stem cell function [85].

Laminopathies are mainly attributed to mutations in the *LMNA* gene [255]. However, more recently two disorders have been linked to mutations in genes encoding B-type lamins: adult-onset autosomal dominant leukodystrophy and (ADLD, OMIM 169500) [183] and acquired partial lipodystrophy (APL, OMIM 608709) [95]. New studies also brought the evidence linking some disease phenotypes with mutations in genes coding for ZMPSTE24 (an enzyme processing A lamins) and various proteins interacting with lamins e.g. emerin, MAN1 (LEMD3), LBR, nesprin-1 and nesprin-2 [147]. Mutations in this gene can cause diseases with highly overlapping clinical features to laminopathies. For example, XL-EDMD caused by mutations in the *EDM* gene coding for emerin, has a very similar phenotype to AD-EDMD caused by *LMNA* mutations.

In recent studies, the mutual influence of emerin and lamin A precursors has been observed [41, 125]. The lack of emerin disturbs the localization of prelamin A, which subsequently leads to perturbation in the mature lamina and chromatin organization, whereas emerin localization is affected by the accumulation of non-farnesylated and farnesylated carboxymethylated prelamin A. In cells from patients affected by EDMD1, in spite of normal lamin A processing, the impaired localization of non-farnesylated prelamin A was observed. In these cells, exogenous expression of emerin restored the normal distribution of lamin A precursor [41, 125]. Similar results were obtained for fibroblasts from EDMD2 patients in whom non-farnesylated prelamin A was mislocalized [143]. In EDMD2 with loss of lamin A protein expression, redistribution of emerin from nucleus to endoplasmic reticulum (ER) occurs [177]. Loss of emerin from the nuclear envelope also occurs in lamin A/C-null mouse fibroblasts [223], which results in aberrant signal transduction. Interactions between emerin and prelamin A may be of great importance for chromatin organization due to their associations with proteins: heterochromatin protein 1 (HP1), LAP2α and BAF, which are known to build a complex that regulates chromatin dynamics and remodeling [41, 125, 143]. This is particularly important during myogenic differentiation when large-scale chromatin reorganization takes place [29].

Phenotypes similar to EDMD are also associated with mutations in the SYNE1 and SYNE2 genes, encoding nesprin-1 and nesprin-2. Nesprins are giant proteins (up to 1 MDa) and contain a C-terminal KASH domain, a small, conservative region interacting with the C-terminal SUN domain of SUN proteins in the perinuclear space. A-type lamins, emerin, nesprins (also known as syne, myne, nuance, enaptin) and SUN proteins participate in the LINC (linker of nucleoskeleton and cytoskeleton) complex, which is responsible for linking the nucleoskeleton with the cytoskeleton [56]. Localization of the nesprins in the NE depends on SUN proteins, which in turn are positioned in the nuclear membrane due to their associations with A-type lamins. One of the nesprin short forms, nesprin-1α, is a product of alternative splicing and is highly expressed in cardiac and skeletal muscles [92, 184, 243]. All mentioned elements of the LINC structure have additional binding partners that enable them to play important roles in the maintenance of cellular function, e.g. control of signal transduction, response to mechanical stress and positioning the nucleus within the cell [56]. It is worth mentioning that only mutations in *LMNA* that cause muscle diseases affect the LINC complex anchoring function of lamin A/C [74].

Three major proteins interacting with lamins (all LAP2 isoforms, emerin and MAN1) are members of a family defined by a 43-residue LEM domain near the N-terminus, which is involved in binding to a chromosomal protein, barrier to autointegration factor (BAF)—a small protein with a role in higher order chromatin structure [249, 255]. BAF mutations cause a progeroid syndrome that is a very similar to laminopathy Hutchinson–Gilford progeria syndrome (HGPS) [196].

Among newly identified NE-associated polypeptides, muscle-enriched A-type lamin-interacting protein (MLIP) [5] and NL-associated NE transmembrane protein (NET39) should be mentioned [132]. MLIP, whose nuclear localization and expression depend on lamin A, is expressed abundantly in skeletal and smooth muscles and may contribute to the mesenchymal phenotypes of laminopathies. MLIP co-localizes with PML protein in PML bodies engaged in the processes such as post-translational modifications, transcriptional regulation, DNA damage responses, and apoptosis [5]. NET39 is also highly expressed in skeletal and cardiac muscles and participates in muscle homeostasis and regeneration [132].

Lamins interact with nuclear F-actin. It has been reported that two EDMD-causing mutations in lamin A significantly reduced such binding, which impact the concentration of the actin in the nucleus and influence processes that require actin (transcription, chromatin remodeling) [218].

To study the role of A-type lamins, several mouse models have been created. Loss of A-type lamins results in growth retardation, developmental defects of the heart, skeletal muscle hypotrophy, decreased subcutaneous adipose tissue and decreased adipogenic differentiation. Such mice develop a form of muscular dystrophy closely resembling EDMD and die from cardiac and skeletal myopathies, usually 8 weeks after birth [119, 223].

Dr. Bonne’s group [10] developed a mice line which carries a missense mutation, causing a histidine-to-proline substitution at position 222 (H222P), originally identified in patients with AD-EDMD. Described animals exhibit overtly normal embryonic development, while for adult individuals scientist observed reduced locomotion, development of cardiac fibrosis, chamber dilation, and hypokinesia with conduction defects. Female homozygotes exhibit pathologies at a later stage and survive longer than males, which die 9 months after birth [10]. Other mice lines described in the literature carry mutation-causing asparagine to lysine substitution at position 195 (N195K) (which causes DCM in humans). Such mice die at an early age due to arrhythmia but also develop severe dilated cardiomyopathy, similar to the human patients [164]. To study the effects of expression of lamin A with the M371K substitution (causes EDMD in humans), a transgenic mice specifically expressing this protein in heart was generated. Noteworthy is the expression of this mutant lamin A in mice, that simultaneously express also endogenous wild-type A-type lamins, caused cardiac pathology (disruption of the cardiomyocytes and abnormal nuclei were observed). Mice were born at lower numbers than that expected, and those born died 2–7 weeks after birth [241].

The knock-in mouse model Lmna(DeltaK32) exhibited striated muscle maturation delay and metabolic defects, including reduced adipose tissue and hypoglycemia leading to premature death. The level of mutant proteins was markedly lower in Lmna(DeltaK32/DeltaK32), and while wild-type lamin A/C proteins were progressively relocated from nucleoplasmic foci to the nuclear rim during embryonic development, mutant proteins were maintained in nucleoplasmic foci. In the liver and during adipocyte differentiation, expression of DeltaK32-lamin A/C altered sterol regulatory element binding protein 1 (SREBP-1) transcriptional activities which was essential for tissue maturation [20].

Cultured myoblasts from *Emd* null mice revealed abnormalities in the cell cycle and differentiation process, but such animals do not develop muscular dystrophy or cardiac conduction defects, and only muscle regeneration revealed some defects [155, 182].

Scientists also developed *Drosophila melanogaster* as a model to study tissue-specific functions of A-type lamins. Expression of the *Drosophila* A-type lamin (lamin C) lacking a head domain, cause numerous defects in larval muscles. Muscle cell nuclei are misshapen and exhibit lamin aggregation, disorganization of the NPC, Otefin, SUN domain protein and the actin-tubulin cytoskeletal network [65, 66]. Interestingly, also in other model organism, *Caenorhabditis elegans*, EDMD mutation cause perturbed body muscle ultrastructure and reduced muscle function, and at the molecular level altered expression of a number of muscle-specific genes [148]. The ΔK46 *C. elegans* lamin mutant animals showed motility defects and muscle structure abnormalities, and revealed alterations in the lamin filament structure at the molecular level [16]. In the zebrafish model, lack of lamin A or expression of progerin affected embryogenesis, increased embryo senescence and aberrant muscle development [116].

The unique feature of laminopathies is that the diseased phenotype appears in humans much later, typically after maturation, than in any other animal model systems studied so far where abnormal phenotype can be detected at the embryonic development stage (fly, zebrafish, frogs) or soon after birth (mice). The disease phenotype in all model system is easily detectable during postnatal development. However, the longer the lifespan the longer is the period of phenotype development. There may be several plausable explanations for this phenomenon.

Laminopathies affect mostly tissues of mesodermal origin—mostly striated muscles. Embryonic development of muscle progenitor cells is governed by morphogens and is less sensitive for other factors. Lamin A/C, emerin, MAN1 and other NL proteins are expressed late in the development when most of muscle progenitor cells were already formed as dermomyotome or myotome. So in laminopathies of muscular dystrophy background, mutated proteins can affect only late stages of prenatal development and postnatal stages of development and regeneration. The relatively short lifecycle of fruit fly, *C. elegans*, zebrafish, frog or even mouse determines their short prenatal and postnatal development—much shorter than in humans. So the disease phenotype appears earlier than in humans.

During late prenatal and postnatal development, muscles increase in size which is correlated with both the increase in size of particular myofiber, but also with the increase in amount of myofibers. This process involves both proliferation of muscle progenitor cells, their maintenance, differentiation of resulted myoblasts, fusion and remodeling of the inter-myofiber connection. During the maturation period, myofibers reach their final, relatively fixed size and extracellular connections. Thereafter, without damage or disease they remain relatively stable with only minimal turnover of original myonuclei. It should be pointed out that most muscular dystrophies in mice models or in humans, although may have different genetic background, have similar phenotypes and timing of its appearance. Thus there is a possibility that the interaction of particular myofiber with surrounding extracellular matrix and neighbouring cells may be affected by the sarcolemma properties, which changes during growth as well [89].

In the following chapters, we attempt to demonstrate the mechanisms and signaling pathways that can affect muscle development during prenatal and postnatal development and regeneration. In addition, we analyze both typical muscular dystrophy laminopathies and HGPS progeria because many of the mechanisms of disease phenotypes discovered during studies on Progeria HGPS development are similar in terms of the involved proteins and pathways. Especially, those involving epigenetic regulation of gene expression by mutated lamins and lamina-associated proteins.

## Prenatal myogenesis and its regulatory mechanisms

Skeletal muscles of the trunk and limbs derive from somites—segments of paraxial mesoderm that form progressively along the anterior**-**posterior axis of the neural tube and notochord. Somitogenesis involves expression of genes involved in Notch and Wnt pathways, morphogen gradients of FGF, retinoid acid and Wnt [19].

Dorsal epithelial compartments of the somites give rise to the structure called the dermomyotome which is divided into epaxial and hypaxial domains. Dermomyotome cells show high, but not uniform, level of *Pax3* and *Pax7* and low level of *Myf5*. The lips of dermomyotome develop into myotome, the primitive muscle structure with committed muscle cells with high level of *MyoD* and *Myf5*. The central part of dermomyotome dissipates and muscle progenitor cells integrate into the myotome [225, 226]. This population of cells gives rise to majority of satellite cells in postnatal muscles.

Pax3- and Pax7-expressing cells within the central dermomyotome give rise to both the muscle satellite cells and the embryonic muscle progenitors, which begin differentiation after activation of the myogenic determination genes—*Myf5* and *MyoD* [88]. However, experiments with Pax7 null mice reveal that Pax7 is not necessary for muscle differentiation since prenatal development of knockout mice is normal with the exception of satellite cells which are progressively lost [201, 202]. This indicates that Pax7 is essential for the maintenance of proper number of satellite cells in muscles by symmetric divisions of muscle stem cells mediated by planar cell polarity (PCP) pathway which probably involves Wnt7a, Vangl-2 and BCL9 [126].

Hypaxial muscles of the body wall originate from ventrally elongated dermomyotome and myotome [51]. Dorsal muscles are generated by epaxial part of the dermomyotome and myotome [186]. Limb muscles and lateral trunk muscles originate from hypaxial domain of dermomyotome.

**Fig. 1:**
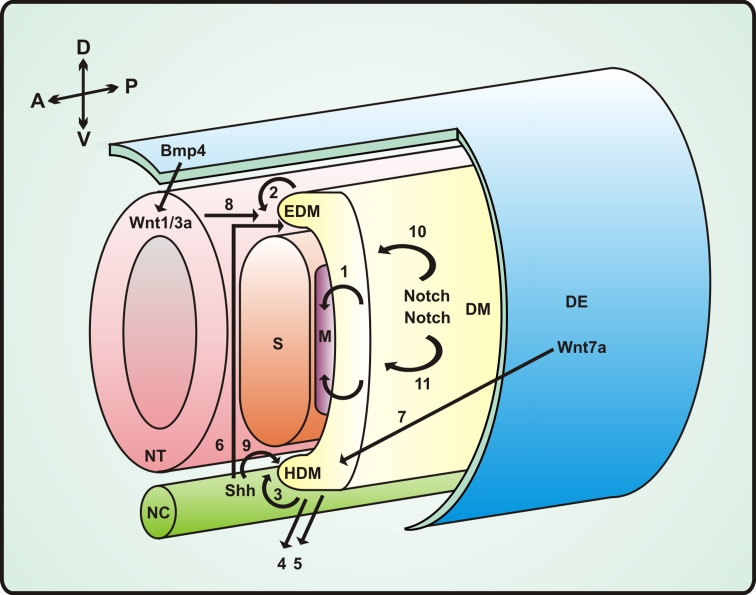
A simplified scheme of signaling pathways and cellular events involved in embryonic skeletal muscle formation. Paraxial mesoderm which forms somites is distributed bilaterally along the neural tube (*NT*) and notochord (*NC*) at the antero-posterior axis. The ventral region of the somite forms the sclerotome (*S*) (which becomes most of the axial skeleton: vertebrae, ribs), the dorsal part of the somite is called the dermomyotome (*DM*). Cellular migration from the dermomyotome gives rise to the epaxial myotome (*EDM*; the muscle of the back) [2] and the hypaxial myotome (*HDM*; body-wall muscles, limb muscles, the diaphragm and the tongue) [3]. The hypaxial dermomyotome is specified by signals from the dorsal ectoderm (*DE*) (Wnt pathway: Wnt7a) and the lateral plate mesoderm (Bmp4) [7]; Wnt7a activates, while Bmp4 inhibits the expression of MyoD. The neural tube (*NT*) and notochord (*NC*) provide signals necessary for epaxial myogenic determination: Wnt1/3a, produced by dorsal NT [8], acts together with Sonic hedgehog (Shh) produced by NC [6]. The epaxial myotome can be identified by *Myf5* expression, while the cells of the hypaxial myotome predominantly express *MyoD*. Muscle progenitor cells delaminate from the dermomyotome (*DM*) and migrate laterally to form the myotome [1] as well as migrate into the limb bud [5], where they continue to proliferate and differentiate later. In limb muscle formation, Shh is also necessary [9]. *A* anterior, *P* posterior, *D* dorsal, *V* ventral, *M* myotome Bmp4 inhibition of the MyoD expression is accompanied by Notch signaling [10] which is responsible for the prevention of early myogenic differentiation of limb buds progenitor cells [11]

The diaphragm, hypoglossal chord and muscles of extremities originate from ventrolateral lip of dermomyotome at the limb level. Head muscles originate from cells of prechordal and pharyngeal head mesoderm [201] (Fig. 1).

Myogenesis is a complex, highly regulated process based on morphogen gradient patterns, genetic network of transcription factors and controlled expression of myogenic regulatory factors. Surprisingly, most of the processes governing the embryonic development of muscles are similar to those regulating postnatal growth and regeneration of muscles.

The morphogenetic events that are involved in the formation of the myotome depends on four main pathways—Bmp, Shh, Wnt and Notch [135] (Fig. 1). Paracrine factors involved in these signaling pathways are delivered by tissues surrounding the differentiating somites such as the neural tube, notochord, surface ectoderm and lateral mesoderm. They supervise the activation or inhibition of different myogenic transcription factors such as Pax3 and Pax7, members of the paired box transcription factors characterized by the presence of a 128-amino acid paired domain that enables sequence-specific binding to DNA [47], which act as upstream activators for myogenic regulatory factors (MRFs) genes transcription. MFRs belong to the family of proteins containing a structural motif basic helix-loop-helix (bHLH) including myogenic factor 5 (Myf5), myogenic determination factor (MyoD), both expressed mainly during early stages of muscle cell differentiation, myogenin and myogenic regulator factor 4 (Mrf4), also known as Myf6 [36]. After fusion, when mononucleated myoblasts become multinucleated myotubes, other muscle-specific proteins such as myosin heavy chains are synthesized.

The most important in formation of dermomyotome and myotome are Wnt proteins. Wnts are conserved lipid-modified signaling proteins involved in many activities during embryogenesis and self-renewal in adult tissues [52]. One of the pathways controlled by Wnts result in the alteration in β-catenin concentration in the nucleus. In the presence of Wnts, β-catenin is bound by the complex which contains, besides Wnts, also frizzled/lipoprotein receptor-related proteins. Due to this complex, β-catenin reaches the nucleus, where it binds to T cell factor (TCF)/lymphoid enhancer factor (LEF) and induces target gene expression. The inner nuclear membrane protein emerin plays a crucial role in regulation of the Wnt-β-catenin pathway, which is responsible for maintenance of stem cell and fate differentiation [231]. Interactions with β-catenin are mediated by a conservative APC-like domain located at the C-terminal end of emerin [146]. However, recent investigations suggest the existence of direct interaction between β-catenin and MyoD with the omission of LEF/TCF [110]. Constitutive expression of endogenous β-catenin or inhibition of its degradation leads to an increase in the subpopulation of Pax7-positive satellite cells and a decrease in the level of cells undergoing terminal differentiation. Reduction of the β-catenin level increases myogenic differentiation of muscle progenitors [188].

Wnt proteins act through canonical β-catenin/TCF dependent pathway and non-canonical pathway through PKC. Wnt1/3 are secreted from dorsal neural tube while Wnt4/6/7a are secreted from surface ectoderm. Wnt1/3 induces Myf5 expression and stimulates Pax3 expression while Wnt4/6/7a stimulates expression of MyoD. Receptor for Wnt7 Frizzled7 (Fzd7) is expressed in hypaxial part of the somite while Fzd1 and Fzd6 (receptors for Wnt1/6) are expressed in epaxial domain. Wnt1 in epaxial domain acts through canonical pathway which leads to expression of Myf5 while Wnt7 (Fzd7) signaling induces MyoD expression through PKC pathway [34]. Thus the differentiation of muscle progenitor cells in hypaxial domain and epaxial domain is regulated diifferently, via different muscle-specific transcription factors.

Another important morphogen—Sonic hedgehog (Shh) is released from notochord and floor plate of neural tube. Together with two other members of the Hedgehog family: Indian hedgehog (Ihh) and Desert hedgehog (Dhh), Shh interacts in mammals with Patched receptor releasing smoothened (the G protein-coupled receptor protein), which in turn regulates target genes transcription through GLI transcription factors [138]. Proper Shh signaling seems to be necessary for maturation of dermomyotomal cells into MyoD/Myf5 positive, committed cells with down regulated Pax3/Pax7 since lack of Shh signaling in myotome results in reduced expression of Myf5 and increases the number of cells with high level of expression of Pax3 and Pax7 which in turn results in blocking of the progression of differentiation.

Shh signaling and canonical signaling through Wnt simultaneously activate the expression of *Myf5* gene due to the presence of regulatory elements in *Myf5* promoter which are recognized by TCF and GLI transcription factors [24]. It should be pointed out that GLI transcription factors can be inhibited by several kinases such as GSK3β, PKA and CK1 which has its further implication also for postnatal muscle cells regulation and shows the complicity of signaling and feedback signaling pathways during muscle development and regeneration.

Interestingly, the abnormally high level of Shh results in induction of sclerotomal marker Pax1 and reprogramming cells into different developmental pathway.

Another important morphogen—bone morphogenic protein (BMP) modify the expression of myogenic genes. BMPs as a subclass of TGFβ proteins signal through serine/threonine kinase receptors and activate transcription through Smad proteins. BMP2/4/7 activates Smad1/5/8 which after complex formation with Smad4 translocates into nucleus and interacts with CBP/p300 and specific transcription factors (e.g. AP-1, bZIP, RUNX, Fox, bHLH). TGFβ and activins act similarly but through Smad2/3 proteins [160]. It is essential to know that BMP signaling and TGFβ signaling also triggers some mitogen activated protein kinase pathways (MAPK) including activation of p38, JNK, ERK1/2 and other intracellular signaling components such as PKC, mTOR and cofilin [58] (Fig. 3d).

In dermomyotome BMP4 increases the level of expression of Pax3 and delays the expression of Myf5 and MyoD which results in expansion of muscle progenitor cells.

Notch signaling is also essential in myogenesis [214]. Notch, a single-pass transmembrane receptor with a large extracellular and a small intracellular domain (N^IC^), during somitogenesis is involved in the determination of somite borders and polarity along the anterior-posterior axis [130]. Notch signaling is based on the interaction of protein Delta/Jagged exposed on surface of “presenting” cell with Notch exposed on surface of “target” cell. Delta1 protein is presented to embryonic muscle progenitor cells by migrating neural crest cells. Notch ligands (DLL1-3, Jag1-2) produced by neighboring cells activate Notch and induce the cleavage off of the N^IC^ domain of Notch. This domain migrates to the nucleus, assembles with CSL factor (CBF1/SuH/Lag-1) and Mastermind (Mam) and activates expression of target genes (Hes family, Myc,p21, Cyclin D3). This in turn alters the expression of different genes [73]. Active Notch suppresses MyoD expression by stimulation of expression of transcription repression protein Hes1. Thus Notch signaling promotes myogenic precursors expansion. Augmentation of BMP signaling results in increase of the number of fetal muscle progenitors and satellite cells in contrast to its repression which has opposite effects [97]. Some reports suggest that the Notch repressive effect on muscle differentiation arises from the ability of the N^IC^ to prevent binding between Maml and its co-activator Mef2 [251] (Fig. 3c). It is worthy to note that the N^IC^ induces expression of MKP1 phosphatase that inhibits p38 kinase involved in the MAP signaling pathway [114].

In order to properly develop the limb muscles, which develop distantly from somites, Pax3-dependent migrating progenitors are required. In mice, depletion of Pax3 causes defects in myogenic progenitor migration, which in turn leads to the suppression of limb muscle development [159]. Limb muscles development starts at the level of limbs in ventral dermomyotome which undergo epithelial to mesenchymal transition (EMT). Long-distance migrating muscle progenitor cells are directed by several factors such as *N*-cadherin, fibronectin, hepatocyte growth factor (HGF) and its receptor c-Met [36].

In myogenesis, the key role play myogenic regulatory factors: Myf5, MyoD, myogenin and MRF4 (Myf6). Myf5 is the first myogenic factor expressed transiently during embryonic development in paraxial mesoderm and later on during the formation of myotome together with MyoD. Myf5 and MyoD may compensate for themselves to certain degree during development but nevertheless at least two populations of muscle progenitor cells are present in myotome: Myf5 positive and MyoD-positive. Both Myf5 and MyoD induce expression of myogenin and MRF4 for myoblast terminal differentiation [77].

Another key role in the regulation of myogenesis play Paired-Homeobox transcription factors: Pax3 and Pax7. Cells in dermomyotome express Pax3 and Pax7. Highest level of Pax3 is in the dorsal and ventral lips while Pax7 is overexpressed in the central domain of dermomyotome. However, Pax3 is expressed in long-distance migrating cells which form initial limb musculature [202]. In mice, depletion of Pax3 causes defects in myogenic progenitor migration, which in turn leads to the suppression of limb and diaphragm muscle development [159]. Current data on Pax3:Pax7 double knockout mutants indicate that Pax3 positive cells are the founder cells (FC) forming initial fibers of muscles in the limb to which secondary fibers and satellite cell pool is added by Pax7 positive cells. Pax3 expression, as well as MyoD, MRF4 and myogenin, is under control of Six1 (sine oculis-related homeobox 1) and Six4 transcription factors. Pax3 and Six proteins participate together in the activation of Myf5 protein promoter.

There is also a feedback mechanism regulating myogenesis involving several microRNAs. The miR206 is upregulated by MyoD targets and inhibits translation and/or degrades mRNAs for Pax3 and Pax7 which facilitates the progression of differentiation.

Proper muscle development requires myoblast fusion. During this process, mononucleated myoblasts become multinucleated myotubes [96]. General rules underlying myoblast fusion are similar in many organisms: FC fuses with the neighboring fusion-competent myoblasts (FCMs). This process is regulated by the asymmetrical distribution of molecules in both the FC and the FCMs [67]. However, it needs to be mentioned that not all invertebrate and vertebrate muscle cells undergo fusion that leads to syncytium formation.

The course of myogenesis is strictly connected with the activity of proteins controlling the cell cycle. For instance, transcriptional activity of Pax3 and Pax7 is modulated due to the interactions with many cofactors, among which members of the retinoblastoma (Rb) family that control the cell cycle exit through modulation of the E2F factors, has to be mentioned [245]. The Rb protein is regulated by cyclin-dependent phosphorylation which comprises the involvement of cell cycle proteins such as cyclin-dependent kinase inhibitor—p21, and cyclin D3 [178]. Hyperphosphorylation contributes to Rb inactivation in proliferating myoblasts. Differentiation signals cause Rb dephosphorylation, which promotes its interaction with E2F transcription factors preventing cell cycle progression and involvement in the synthesis of late skeletal muscle differentiation markers—muscle creatine kinase and Mrf4 [61, 62] (Fig. 3l). Inactivation of the E2F transcription factors occurs by its binding to dephosphorylated Rb protein in complex with LAP2α and lamin A. Due to the interaction, the cell remains stalled in the G1 phase. Rb is also engaged in myoblast fusion, muscle fiber formation and the maintenance of terminally differentiated muscles [104].

Myogenesis is also regulated by associations between bHLH factors and Mef2. The activity of these factors is regulated by their interactions with histone deacetylase (HDAC), histone acetyltransferases, and the SWI/SNF chromatin remodeling complexes [151]. During differentiation, muscle-specific genes are activated by the release of HDAC from their promoters. For example, dephosphorylated Rb is able to bind HDAC, which allows it to take part in MyoD acetylation (by CBP–CREB-binding protein) by releasing it from HDAC1. This process is modulated by phosphorylation and recruitment of histone acetyltransferase (e.g. myogenin is switched on by the release of HDAC from its promoter through phosphorylation and recruitment of histone acetyltransferase via MyoD and Mef2) [151]. MyoD, together with MEF2, induces expression of the genes responsible for cell cycle withdrawal and terminal differentiation [104] (Fig. 3k).

The enzymes mentioned above, which modify chromatin, participate in the regulation of myogenesis, due to the formation of complexes with transcription factors that recognize a specific DNA sequence located at gene regulatory regions. These complexes influence gene expression via control of the spatial organization of the chromatin [210].

Due to many studies, it is currently known that proper muscle development also requires engagement of the NE proteins (A-type lamins, emerin, LAP2α, MAN1) which form a functional scaffold for different transcription factors and create complexes that modulate activity of muscle differentiation factors e.g. myogenin and cell cycle controlling proteins such as Rb [15, 72, 145].

Overexpression of mutant lamin A (R453) causing AD-EDMD disrupts differentiation of C2C12 myoblasts correlates with decrease in the expression of myogenin and with accumulation of hyperphosphorylated Rb protein. These abnormalities lead to deficiencies in interactions necessary for cell cycle arrest and myoblast fusion necessary for the formation of mature myotubes [72]. Also the NE proteins’ expression and distribution is changed in the presence of the other AD-EDMD-causing lamin A mutant (W520S). During normal myoblast differentiation, the following changes are observed: increase in lamin B2 expression, decrease in LAP2α expression, reduction of lamin A and C solubility and their redistribution. The presence of mutant lamin A which accumulates in the nucleoplasm results in inhibition of myoblast differentiation caused by disruption of underphosphorylated Rb/LAP2α complexes [145], which are formed under normal conditions when Rb dephosphorylation is necessary for the onset of differentiation (Fig. 3k). Also reduction of lamin A or emerin levels is correlated with the decrease in expression of four important differentiation factors: MyoD, desmin, Rb and M-cadherin. Moreover, because in lamin A knockout myoblasts expression of Pax7 and Pax3 is not disrupted [75], it seems that impaired differentiation results directly from a lamin A deficiency.

## Postnatal myogenesis and its regulatory mechanisms

Postnatal muscle growth is associated with the activation and differentiation of embryonically derived myoblasts present in the satellite cell pool [179]. The presence of Pax7 + cells which can contribute to muscle growth and regeneration in juvenile and adults was recently confirmed in various species [46]. The role of Pax7 in postnatal myogenesis seems to be consistent with the regulation of Myf5 expression in myoblasts derived from satellite cells by binding to a paired domain motif-containing enhancer [37]. However, recently obtained results about Pax7 activity are equivocal and inconsistent. For example, on the one hand Pax7 stimulates myogenesis via up regulation of Myf5 in a histone methyltransferase complex dependent manner, but on the other hand Pax7 contributes to the suppression of myogenesis by MyoD inhibition [149].

A growing number of studies indicate that activities of different myogenic regulatory factors are developmental stage-dependent and may be substituted by various proteins (among which not all have been identified) that mediate reciprocally connected or completely independent signaling pathways. For example, Pax7-depleted muscles derived from juvenile and adult individuals differ in phenotypes. This is due to Pax7 involvement in myogenesis during the juvenile period when progenitor cells make the transition into quiescence [128]. Furthermore, although depletion of Pax7 is not substituted by Pax3 activity, compensation for its loss by other related proteins in the adult, but not in the embryo or the pups, cannot be excluded [239]. Involvement of bHLH factors in postnatal myogenesis was also investigated. For example, postnatal (contrary to prenatal) inactivation of myogenin does not have dramatic effects on muscle activity except for body size, which is reduced [112]. This suggests its minor role in the maintenance of adult muscle homeostasis.

Adult myogenesis with reference to muscle regeneration via satellite cell activity is discussed in the next section.

## Regulation of prenatal satellite cells development, maintenance and adult muscle regeneration

Satellite cells originate from a population of undifferentiated stem cells in the embryonic dermomyotome [88]. All satellite cells in limb muscle originate from Pax3 + cells of the hypaxial dermomyotome [213]. The presence of Pax3 is necessary for mesenchymal epithelial transition factor (c-Met)—tyrosine kinase receptor expression which is engaged in delamination and enables proliferation of muscle precursors as well as their migration from dermomyotome into the limbs [22]. Pax3 modulates expression of c-Met via the interactions with regulatory sequence in the *c*-*Met* promoter [38].

Pax7 is one of the characteristic markers of satellite cells. Its presence was observed within two subpopulations of adult muscle stem cells: Myf5-/Pax7+ (true muscle stem cells) and Myf5+/Pax7+ (committed progenitors) [117]. Both types of cells are generated during asymmetric division. Former group is responsible for self-renewal of the satellite cell population, latter takes part in terminal differentiation program leading to formation of new muscle fibers [117]. Muscle progenitor cells express other genetic markers: M-cadherin—a cell surface adhesion protein that probably plays role in terminal muscle differentiation, CD34—surface marker of quiescent satellite cells [254] and myostatin—secreted member of TGFβ family that ensures the resting state of satellite cells.

At the end of fetal life, myogenic progenitors known as satellite cells relocate at muscle fibers. These mononucleated, quiescent stem cells are embedded within the basal lamina and during postnatal growth or in case of injury or pathological conditions (e.g. dystrophies) they are activated, start to differentiate and fuse to form new muscle fibers.

**Fig. 2:**
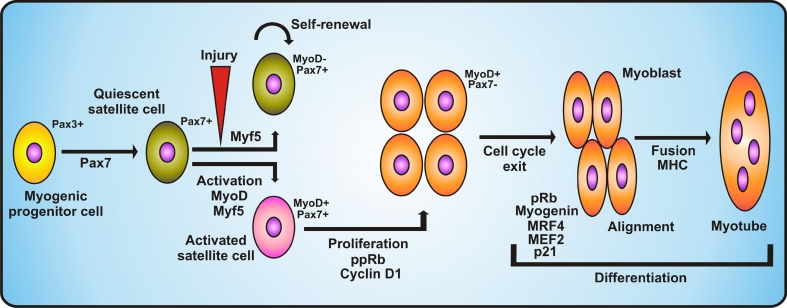
Regulation of proliferation and differentiation of myogenic progenitor cells. The diagram demonstrates the stages of myogenic progenitor cell transformation into quiescent satellite cells and upon activation/injury into activated satellite cells. Activated satellite cells expressing MyoD and Pax7 undergo proliferation stimulated by hyperphosphorylated Rb protein and cyclin D1. Proliferated satellite cells undergo cell cycle arrest correlated with the loss of Pax7 expression. Further differentiation into myoblasts is induced by pRb, myogenin, MRF4, MEF2 and p21. After alignment, myoblasts undergo fusion into myotubes. See main text for detailed description

Satellite cells are capable of self-renewal due to their capacity for asymmetric division, which gives rise to two nonidentical daughter cells. One of these cells remains quiescent while the other is activated and undergoes myogenic differentiation. Asymmetric division is accompanied by asymmetric segregation of Numb protein (Notch inhibitor), which is inherited by the daughter cell that becomes an FC and takes part in muscle formation, whereas the second sibling cell suspends its differentiation process [117]. The subpopulation of satellite cells, which after proliferation downregulates Pax7, differentiates, whereas cells which maintain Pax7 expression and inhibit production of MyoD return to a quiescent state [117] (Fig. 2). Recent studies indicate that the persistent contact with the basal lamina of the myofiber allows satellite cells to retain their stem cell status. Loss of contact leads to the activation of a differentiation program which is preceded by Myf5 expression. Moreover, active Bmp signaling occurs in proliferating satellite cells during regeneration, which suggests that this pathway is crucial for activated satellite cells in adults [240].

Since the presence of Pax7 is essential in myogenesis and satellite cells maintenance, specification, self-renewal and expansion (also in adulthood) [181] downregulation of Pax7 by myogenin promotes myogenic differentiation. Similar to Pax7, which is expressed by all satellite cells and acts as a survival factor, Pax3 is found in adult satellite cells. However, its presence is restricted to cells from the diaphragm and a subset of lineages of forelimb muscles [117].

**Fig. 3:**
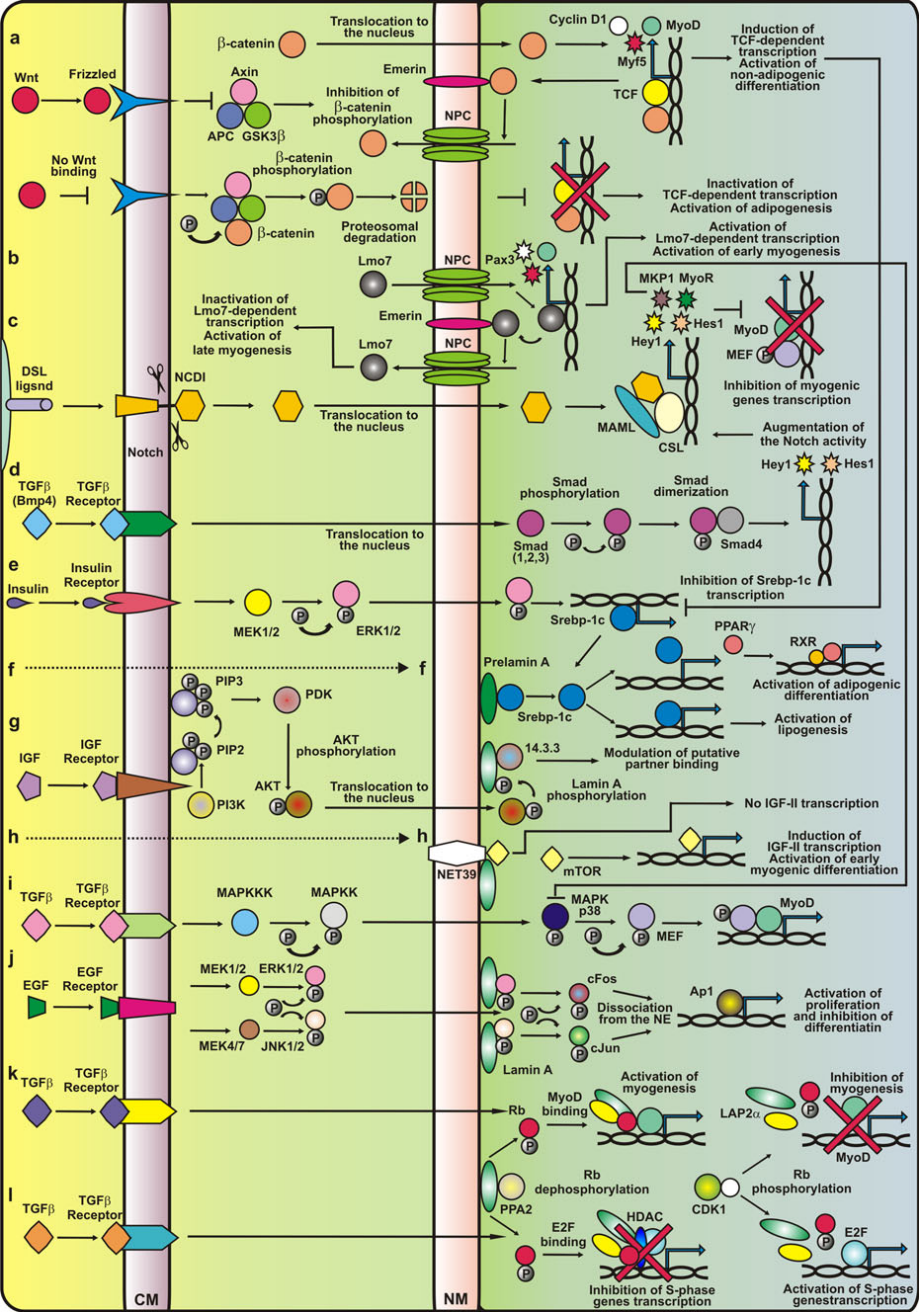
The molecular background of major signaling pathways responsible for proper development of muscles and muscle stem cells in normal tissues and tissues affected by laminopathy. Only major signaling pathways are shown. In the case of Wnt and Notch signaling pathways, only essential fragments of pathways for muscle tissues are shown. In the case of TGFβ-related pathways, only four different pathways are shown as an example of possible interactions. **a** Wnt/β-catenin signaling is activated by ligands of the Frizzled receptor, which triggers a signaling cascade resulting in the displacement of the multifunctional kinase GSK3β from the APC/Axin/GSK3β complex. Free GSK3β is not able to phosphorylate β-catenin. Hypophosphorylated β-catenin accumulates in the cytoplasm and is translocated to the nucleus, where it can be immobilized in nuclear envelope (NE) by emerin binding (inactive β-catenin) or can translocate to the nucleoplasm, where it activates its target genes via TCF/LEF (T cell factor/lymphoid enhancer factor) transcription factors. Mutations in emerin could change β-catenin cellular localization and thus influence its activity. In the absence of Wnt, cytoplasmic β-catenin is constantly phosphorylated by GSK3β in a protein complex, which leads to β-catenin ubiquitination and proteasomal degradation. **b** For proper myoblast differentiation, LIM-domain only 7 (Lmo7) is needed. This is a transcription factor that regulates Pax3 expression. Emerin binds to and inhibits the activity of Lmo7. **c** Notch binding to ligand elicits several steps of cleavage; thus the intracellular domain (NICD) of this protein is released and translocated into the nucleus where it forms a complex with the DNA-binding CSL transcription factor and the transcriptional co-activator Maml. This complex induces the expression of *Hes1* and *Hes5* genes. The Notch repressive effect on muscle differentiation arises from intracellular domain of Notch (NICD) ability to prevent binding between MAML and Mef2. **d** Bmp4 acts by augmentation of Notch-dependent transcription, inducing phosphorylation of Smads, which after heterodimerization with Smad4 leads to transcription stimulation of target genes including expression of Hes1 and Hey1 molecules. **e**, **f** SREBP1 interacts with pre-lamin A, but not with the mature form of this protein. SREBP1 is a transcription factor which regulates lipogenesis, and plays a role in the transactivation of the PPARγ promoter (**f**). Following activation by retinoic acid, PPARγ forms heterodimers with RXR (retinoid X receptor) and modulates gene transcription. Mutations of lamin A cause the retention of SREBP1 at the NE and decrease the SREBP1 level in the nucleoplasm, which reduces the expression of PPARγ and other genes. **g**, **h** Lamin A is phosphorylated on the S404 residue by Akt/PKB kinase downstream of the phosphoinositide-3-kinase (PI3 kinase) signaling pathway. The phosphorylation of lamin A might modulate interaction with protein partners, impair lamin polymerization or promote association with the 14.3.3 protein. Lamin mutations can inhibit S404 phosphorylation and lead to changes in NE stability. **j** The MAPK kinases activate extracellular signal-regulated kinase (ERK) and c-Jun NH[2]-terminal kinase (JNK). Active, phosphorylated ERK1/2 and JNK kinases are translocated to the nucleus where they interact with A-type lamins at the nuclear periphery and phosphorylate c-Fos and c-Jun, causing their release from the NE, thus allowing the transcriptional activation of responsive genes. Lamin A mutations, inhibit the binding of mutated lamin A/C proteins with ERK and JNK kinases, cause their release from the complex with lamins and in consequence increase the activity. **k**, **l** Lamins could modulate TGF-β-dependent signaling through interaction with protein phosphatase 2A (PP2A). PP2A dephosphorylates pRb, and dephosphorylated Rb is able to bind MyoD. The MyoD-Rb-lamin A-LAP2α complex induces the expression of genes associated with myogenesis. Cdk4 phosphorylates MyoD, releasing it from the lamina-LAP2α-pRb complex, leading to the inactivation of myogenesis. This creates an opportunity for pRb to take part in the regulation of cell cycle progression and proliferation. In the absence of wild-type lamin A/C, PP2 is unable to dephosphorylate pRb in the lamin A-LAP2α complex. Phosphorylated pRb does not bind MyoD, and this leads to inactivation of myogenesis and a decrease in MyoD and pRb levels. See main text for adetailed description

Interactions between the Wnt and Notch signaling pathways play a crucial role in the maintenance and activation of muscle satellite cells through canonical pathway [28] (Fig. 3a, c). β-catenin, located in a multiprotein complex at the plasma membrane, is also engaged in myoblast fusion by binding with cell adhesion complexes containing cadherins [78].

In adult satellite cells, the activity of the Notch signaling pathway prevents their premature differentiation. Muscle injury contributes to Notch activation [55]. This leads to enhanced proliferation and satellite cell expansion. Subsequent inhibition of Notch signaling precedes activation of the canonical Wnt signaling pathway that plays a role in late stages of muscle regeneration during which differentiation into myoblasts of previously multiplied cells must occur [28]. However, it should be noted that some researchers obtained inconsistent data according to which the activation of β-catenin-dependent Wnt signaling is associated with the activation of satellite cell self-renewal [188].

The Notch pathway links with Wnts signaling via intracellular signaling glycogen synthase kinase 3β GSK3β. Canonical Wnt signaling in contrast to the Notch pathway inhibits kinase GSK3β activity [126].

One of the NE protein, emerin, is also engaged in regulation of the Wnt-β-catenin pathway due to the interactions with β-catenin which are mediated by a conservative APC-like domain located at the C-terminal end of emerin [146].

The non-canonical Wnt pathway is also engaged in regulation of satellite cell self-renewal. During muscle regeneration, Wnt7a together with its receptor Frizzled7 (Fzd7) through the mediation of the planar cell polarity pathway stimulates the symmetric expansion of the satellite cell pool [126].

Inappropriate Wnt signaling disrupts muscle regeneration, leading to disease [28]. It is particularly interesting in the light of recent reports that in aged muscles a high level of Wnt signaling in quiescent and early activated satellite cells contributes to activation of the fibrogenic or adipogenic program [55]. Disruption of these signaling pathways by EDMD-causing mutations could also lead to misregulation of muscle repair and thus to the development of dystrophic phenotypes [212].

In muscles of patients suffering from different muscular dystrophies the regenerative potential is lower than that in the healthy tissues [55, 136, 162]. Satellite cells do not contribute properly towards the regeneration of diseased muscles [1, 207]. Abnormalities of satellite cells may be induced by mutations in genes coding for lamin A and emerin which leads to the weakness of adult myofiber regeneration, causing e.g. EDMD progression [145, 188].

This has been observed in satellite cells from patients with AD-EDMD or LGMD1B. In the diseased adult muscle progenitors, disorganization of chromatin was accompanied by increased numbers of Pax7-positive nuclei and a reduced number of MyoD-positive nuclei [185]. These results are in perfect agreement with previous observations indicating that reciprocal inhibition between the transcription factors Pax7, MyoD and myogenin modulates satellite cell fate decisions probably through typical cell cycle regulation mechanism associated with lamin A, LAP2α and pRb complexes [83, 84, 174].

Mice lacking emerin also exhibit defects in muscle regeneration connected with abnormalities in the cell cycle [155]. Observed changes are associated with the disturbances of transcriptional pathways dependent on Rb protein and MyoD. Compared to normal myogenic cells, *Emd* null myoblasts showed prolonged hyperphosphorylation of Rb [15].

It is noteworthy that other types of adult muscle stem cells (mesenchymal stem cells) are affected by abnormalities in the Notch and Wnt signaling pathways caused by mutations in genes coding for A-type lamins [212] or ZMPSTE24 [70]. These disorders lead to a laminopathy called Hutchinson–Gilford progeria syndrome (HGPS) [69, 237] or a more severe restrictive dermopathy (RD) [175], rare childhood diseases characterized by premature ageing, alopecia, wrinkled skin, loss of subcutaneous fat, joint abnormalities and early death (in the neonatal/perinatal period in the case of RD) [69, 70, 163, 212, 237].

## Side population (SP) cells—another muscle stem cell population

Muscle regeneration is mainly conjoined with the satellite cells. However, some studies assume that another stem cell fraction known as side population cells (SP cells) which are present in skeletal muscles (termed skeletal muscle SP cells) are involved in this process [90, 91]. Although experiments undertaken within recent years has generated valuable data about SP cells localization, embryonic origins and activity, their exact role in muscle regeneration remains elusive.

Side population cells are a group of hematopoietic stem cells belonging to bone marrow stem cells (BMSCs) and are localized in the interstitial connective tissue of the skeletal muscle close to the blood vessels. They were first identified due to their ability to exclude Hoechst 33342 dye [82].

The SP cells can be considered as a potential tool in medical treatment of muscle diseases due to their qualities that are considered indispensable for muscle regeneration. When injected into muscle of lethally irradiated mice, SP cells, besides causing recovery of the hematopoietic system, are able to differentiate into myoblasts [91]. Moreover, it was also reported that SP cells delivered to injured muscles contribute to their recovery by undergoing differentiation into satellite cells and can take up a position in their niche [150]. SP cells may be of great interest to scientists elaborating therapeutic strategies for dystrophic patients due to their ability to be delivered systemically through the circulation, which may facilitate repair of diseased muscles [13].

The majority of limb muscle SP cells, similar to limb muscle satellite cells, originate from cells expressing Pax3 and their migratory derivatives located in the hypaxial somite. SP cells with somitic origins display greater myogenic potential than SP cells which are derived from other regions [213]. Non-somitic sources from which a small fraction of SP cells may arise are bone marrow, hematopoietic stem cells, and endothelial cells. This diverse provenance is reflected in SP cell marker heterogeneity [213].

The SP cells are a heterogeneous population among which CD45+ and CD45− fractions can be distinguished. These two groups indicate myogenic potential but only SP CD45+ includes cells with hematopoietic potential [11]. Due to the identification of the novel marker CD31, further characterization of SP cells was made possible [234]. This allowed the subdivision of the known pool of SP cells which appeared to be more heterogeneous with respect to their differentiation potential. CD31+/CD45− SP cells are the main sub-fraction in non-regenerating muscle, whereas not so numerous CD31−/CD45− SP cells reveal greater myogenic potential in vitro as well as in vivo. Furthermore, in case of an injury they become the prevailing fraction and express regulatory genes which are crucial during muscle regeneration as well as muscle markers [234].

Skeletal muscle SP cells are distinct from bone marrow side population cells and satellite cells and exhibit a specific marker, stem cell antigen-1 (Sca-1), which is not present on adult satellite cells [161]. It has been shown that in Pax7 null mice the fraction of satellite but not the side population cells was reduced [215]. This suggests that SP cells, in contrast to satellite cells, arise independently of Pax7.

SP cells reveal their myogenic potential (inter alia by Pax7 expression) when they are cultured together with C2C12 myoblasts [11]. SP cells may also be related to (at least some subset of) satellite cells, as was demonstrated in the studies undertaken by Tanaka and co-workers [227]. In their research, SP cells also expressed, besides Sca-1, syndecan-4 (a marker of satellite cells) and Pax7. Moreover, newly identified cells reside beneath the basal lamina in the satellite cell position and were able (after direct injection) to regenerate diseased muscles. Because of these features, the term satellite-SP cells has been proposed [227].

## Mutations in laminopathies affect myogenesis, postnatal muscle growth and muscle regeneration by various mechanisms

Current data indicate that lamins and lamina-associated proteins of NE may affect most of the signaling pathways involved in myogenesis during both embryonic development and postnatal muscle growth and regeneration. It seems that similar signaling pathways govern the muscle cell specification in dermomyotome and myotome during embryogenesis as during postnatal muscle growth. Thus, the major unanswered question is when do lamins and lamina-associated proteins start to be expressed during embryogenesis and what interacting protein partners are also present in particular tissue? The recent discovery that lamin A/C mRNA can be detected in mice during embryogenesis at the stage E11 in heart, outflow tracts, dorsal aorta, liver and somites [119], proves that lamins and lamina-associated proteins can indeed affect embryonic myogenesis [205]. Although, two animal models of lamin A/C knockout [119, 223] differ in severity of phenotype, together with many mice laminopathy models, they provide the same message, that lamins and lamina-associated proteins affect myogenesis and muscle regeneration.

## Cell cycle control and regulation of Rb

Cell cycle control and regulation of Rb location and phosphorylation status seems to be the most obvious mechanism by which lamins and lamina-associated proteins may affect myogenesis and regeneration. The link between pRb, LAP2α, lamin A and muscles was established by detailed analyses of effects caused by loss of A-type lamin expression and provided the first direct evidence that the location of lamins within the nucleus is important for cellular differentiation [72, 145]. Observed abnormalities are correlated with decrease in the expression of myogenin and with accumulation of hyperphosphorylated Rb protein. Moreover, in the presence of mutant lamin A (R453W), myoblasts are able to express proliferation markers but they display deficiencies in interactions necessary for cell cycle arrest and they do not fuse to form mature myotubes. These data led to the hypothesis that the transition of myoblasts into myocytes depends on a functional scaffold built by the lamins and their partners [72]. During normal differentiation, the level of lamin B2 increases while LAP2α expression decreases, lamin A and C proteins are redistributed and less soluble. In AD-EDMD-causing lamin A mutants (e.g. W520S) inhibition of myoblast differentiation is observed by preventing reorganization of NL. The presence of mutant lamin which accumulates in the nucleoplasm results in the disruption of underphosphorylated Rb/LAP2α complexes [145], which are formed in normal conditions when dephosphorylation of Rb is necessary for the onset of differentiation. Disruption of mentioned complexes is correlated with loss of Rb synthesis. Moreover, expression of exogenous lamin A causes retention of LAP2α in the insoluble complexes within the nucleoplasm [145] Loss of LAP2α in mice results in delay in satellite cells differentiation and postnatal fiber differentiation most probably due to the lack of ability to form nucleoplasmic LAP2α-lamin A complexes which play crucial role in proper timing of reprograming of muscle progenitor cells from proliferation to differentiation [83, 84, 174] (Fig. 3k).

Beside lamin A, emerin also is involved in EDMD development. Myoblasts with decreased emerin expression show changes in differentiation phenotype similar to those observed in *LMNA* knockout mice. In both cases, reduced level of lamin A or emerin is correlated with decrease in the expression of four important differentiation factors: MyoD, desmin, Rb and M-cadherin. Reduction of cytoplasmic protein, desmin and MyoD as a result of lamin A knockout shows their importance in muscle formation, because restoration of their normal level increases the differentiation potential in lamin A null myoblasts. Moreover, these findings suggest that reduction of desmin, which acts as a mediator between the cytoplasm and the nucleus, could contribute to deficiency in communication between these two compartments [75].

The Rb protein, which is regulated by cyclin-dependent phosphorylation, seems to play a crucial role both in proliferation of activated myoblasts and in the cell cycle exit. Hyperphosphorylation, specifically pocket C phosphorylation, contributes to Rb inactivation in proliferating myoblasts. Differentiation signals cause Rb dephosphorylation, which promotes its interaction with E2F transcription factors preventing cell cycle progression [61] (Fig. 3l).

One explanation of the pathological mechanism leading to the development of dystrophic phenotypes assumes that mutations in genes coding for the NE components trigger destabilization of the transcriptome in differentiated cells. The transition from myoblast proliferation to cell cycle exit is correlated with differences in expression of important lamina components: lamin B1, LAP1 and LBR levels decline, whereas those of lamin A/C, lamin B2 and LAP2 increase [15]. Disruption of interactions between the NE elements lamin A and emerin, Rb protein and MyoD cause failure of critical steps during myoblast exit from the cell cycle. Abnormalities are induced by incorrect timing of phosphorylation and acetylation of these proteins [155]. As mentioned above, hypophosphorylated Rb takes part in acetylation of MyoD (by CBP–CREB-binding protein) by releasing it from HDAC1 [197]. MyoD, together with MEF2, induces genes responsible for cell cycle withdrawal and terminal differentiation [104] (Fig. 3k). Loss of lamin A and emerin may disrupt muscle function and regeneration differently. *LMNA* null mice and myoblasts, differ from *EMD* null mice and myoblasts and pathways involved in muscle regeneration are affected differently [14, 155].

Recent studies have allowed the molecular basics of symptoms typical for EDMD to be explained. The other phenotypes (including lipodystrophy and cardiomyopathy) associated with different mutations in the *LMNA* gene might also be caused (at least partly) by alterations in the cell cycle timing and differentiation. These two processes are critical for the development of various tissues, including muscle, heart and adipocyte, and remain under strict control of the pocket proteins family: Rb, and Rb-like proteins RBL-1 (p107) and RBL-2 (p130) [115]. Regulation of the development of different tissues may require interaction between Rb/lamin A/LAP2α complexes and various tissue-specific transcription factors. This is consistent with the discovery of another lamin A binding partner, sterol regulatory element binding protein (SREBP), a transcription factor important for adipogenesis [15, 134] (Fig. 3e, f). The abnormal mechanisms of cell cycle regulation associated with laminopathies involving abnormal signal transduction are discussed below.

## The involvement of lamins and lamina-associated proteins in signal transduction

Nuclear envelope and nuclear lamina proteins including A-type lamins are engaged in modulation of the MAPK, Wnt-β-catenin, TGFβ/Smads, Akt, mTOR, Shh and Notch signal transduction pathways. Misregulation of these signal cascades is often observed in several laminopathies and may lead to an explanation of their pathogenesis and etiology.

## MAPKs

Among signaling pathways controlling cell proliferation, growth and differentiation that influence muscle formation, the MAPK-ERK1/2 cascade should be mentioned (Fig. 3j). Activation of MAPK signaling kinases induces a kinase cascade, including ERK5, p38, JNKs and ERK1/2/1c, which phosphorylates target effectors (cytoplasmic or nuclear) and transcription factors [191, 192]. In satellite cells, inhibition of p38 disrupts both the activation of quiescent satellite cells and terminal differentiation [107]. This is consistent with the fact that activation of some target genes of MyoD depends on p38 kinase activity.

A-type lamins are a binding partner for the transcription factor c-Fos, which together with c-Jun participates in formation of AP1—the transcription factor activating protein mediating cell proliferation and differentiation [80]. Please note that c-Fos is a target of three signal cascades: ERK5, p38 and ERK1/2/1c while c-Jun is activated by p38 cascade so these proteins are important participants in all signaling pathways that belongs to the MAP kinase family. Lamins sequester c-Fos to the NE. This allows cells to remain in a quiescent state. The activation of ERK1/2 causes c-Fos release and turns on the transcription of target genes necessary for cell spreading [80]. This is consistent with the fact that lamin A null cells exhibit increased proliferation [105]. Furthermore, similar observations have been made for Rb protein. In this case, cell proliferation was also enhanced by lamin A depletion [187].

For proper muscle differentiation, inactivation of ERK1/2 must occur. Patients with EDMD2 exhibit increased activation of ERK1/2. Analysis of gene expression profiles from mouse models of EDMD (H222P and emerin-null) revealed abnormalities in the MAPK pathway, which is involved in the development of cardiomyopathy. Moreover, hyperactivated ERK1/2 has been observed in *LMNA* knockout HeLa cells, C2C12 myoblasts and AD-EDMD model mice [167, 170].

## TGFβ/BMP

TGFβ/BMP/Activin signaling pathways act through Smad1/5/8 proteins or through Smad2/3 proteins. BMP signaling and TGFβ signaling also triggers some mitogen activated protein kinase pathways (MAPK) including activation of p38, JNK, ERK1/2 and other intracellular signaling components such as PKC, mTOR and cofilin [58] (Fig. 3d). In normal muscle cells and satellite cells, the presence of lamin A, emerin, NET25 and MAN modulate the signals from MAPK and TGFβ pathways (also mTORC1 pathway). When these proteins are mutated or absent, over activation of effector transcription factors occurs [103]. Emerin and NET25 have partly overlapping functions in modulation of ERK1/2 signal transduction pathway while MAN1 affects both ERK1/2 and TGFβ signaling (the latter through sequestering of Smad proteins) [137, 166, 168, 169, 171, 252]. Mutations in the gene coding for MAN1 lead to hyperactivation of this pathway, which in turn promotes osteoblast differentiation and has implications for muscle and cardiac fibrosis {Capanni, 2009 #5923) [235].

A-type lamins interact with Smads 2/3, which leads to the inhibition of TGFβ/Smad-dependent transcription. The underlying cause of this phenomenon is dephosphorylation of Smads via PP2A kinase, which is promoted by lamins [167, 235]. EDMD-causing lamin A mutations as well as absence of lamin A contribute to the alterations in Smads phosphorylation. Indeed, impaired TGFβ/Smad signaling has been linked with muscle fibrosis in lamin A knockout mice [235] (Fig. 3d).

## mTOR

mTOR pathway integrates inputs from five different extracellular signals: growth factors, genotoxic stress, energy status, oxygen and amino acids. mTORC1 activity is inhibited by TSC1/2 proteins which in turn are inhibited directly by Akt, RSK1 and ERK1/2 kinases and IKKβ keeping mTORC1 active. GSK3β kinase which branches the signal from Wnt, activates TSC1/2 thus inhibiting mTORC1 signaling. Active mTORC1 activates SREBP1 and PPARγ (Fig. 3h). Interaction between insulin-like growth factor II (IGF-II) and the Ser/Thr kinase mammalian target of rapamycin (mTOR, required for IGF-II expression) participates in the early stage of myogenesis [68]. This interplay is mediated by NET39, which negatively regulates myogenic differentiation due to its ability to bind mTOR [132]. This in turn leads to the repression of IGF-II induction. Since the knockout of NET39 (contrary to its overexpression) increases mTOR activity and thereby IGF-II synthesis during early myoblast differentiation, it is thought that NET39 may sequestrate mTOR at the NE and act as a part of negative feedback for the regulation of myogenesis (Fig. 3h). Its synthesis during myogenesis is regulated by early myogenic regulatory factors such as MyoD and MEF2. NET39 is highly expressed after myoblast differentiation and in mature muscle, which probably reflects its participation in muscle homeostasis [132] (Fig. 3h). It is possible, that one of the signaling proteins in muscles and myoblasts transmitting signal between mTOR, MAPs and IKKβ may be S100B protein [233].

Recent analysis of properties of HGPS cells and progerin indicated that lamin A is involved in pathways regulating cellular response to stress and oxidative stress and the effect of rapamycin on partial restoration of wild type properties of HGPS cells [87]. The molecular background of the effect of rapamycin on HGPS cells may be based on both mTORC1 and mTORC2 complexes. Active mTORC1 inhibits insulin and IGF signaling and via different pathway activates, together with PGC1 and YY1, transcription of genes involved in mitochondrial proliferation and oxidative metabolism. mTORC2 complex acting through Akt and probably 14-3-3 protein inhibits FOX01 and FOX03a transcription factors regulating stress resistance, apoptosis and metabolism [123, 124] Recent data indicated that mTORC1 directly stimulates SREBP-1c and uncouples lipogenesis from gluconeogenesis [123].

## Akt signaling

Lamins are linked with PI3 kinase/Akt signaling, which is necessary for myocyte maturation [246]. Insulin stimulation seems to play a role during muscle development and regeneration. A-type lamins are phosphorylated at their N-terminal end by Akt kinase in response to insulin. This modification is impaired in laminopathic muscles (EDMD and LGMD1B) [43, 45] (Fig. 3g). Akt phosphorylates lamin A also at the Ser404 residue, which is reported to be a putative binding site for 14.3.3 proteins [43]. Moreover, phosphorylation at this residue has been involved in positioning of human lamin A [129]. Members of the 14.3.3 family are responsible for modulation of the G2/M phase transition. They also probably interact with A-type lamins during mitosis [6]. Indeed Ser404 is identified as phosphorylated on soluble lamin A, which seems to support this thesis. Phosphorylation of lamin A at Ser404 via Akt kinase may influence lamin A stability and polymerization. This, in turn, may have an effect on lamin A ability to interact with other factors playing a role in modulation of various signaling pathways [43].

## Adipogenic program

The central step in the activation of adipogenic program is expression of peroxisome proliferator-activated receptor gamma (PPARγ), which is the main adipogenic transcription factor and strictly depends on the β-catenin level. Standing activation of β-catenin inhibits PPARγ expression, which results in suppression of adipogenesis. Because accumulation and stability of β-catenin depend on emerin (and A-type lamins), it was suggested that emerin level could be the main factor modulating the balance between β-catenin and PPARγ, hence influencing adipogenesis [231]. These observations suggest that there is a link between emerin mutation and the progressive replacement of skeletal muscle fibers and cardiomyocytes with fatty fibrotic tissue in patients with X-linked EDMD [7, 231] (Fig. 3f).

It should be pointed out that hedgehog signaling pathway when upregulated can induce reprogramming of myogenic progenitor cells through activation of Pax1 and inhibition of expression of Pax3 [25–27]. This switches on the sclerotome development programme. Postnataly hedgehog pathway may be affected by PKA, GSK3β and CK1 kinases.

## Modulation of chromatin organization and gene expression

Peripheral regions of the nucleus (with some exceptions) are thought to be transcriptionally inactive. Lamina-associated domains (LADs) mainly created by lamins include gene-poor regions and help in preservation of the repressive environment. Components of the NL other than lamins (e.g. LBR, emerin, human LAP2β) due to their participation in LAD formation also play a role in chromatin silencing [139, 217]. As indicated recently, *Xenopus laevis* XLAP2 isoforms may also contribute to the maintenance and the anchoring of chromatin domains to the NE and may participate in formation of lamin B or lamin A microdomains [49]. This seems very plausible since incorporation of newly synthesized lamins A and B is not uniform and is accompanied by formation of separate microdomains [76, 217]. Proteins of the INM such as LBR and XLAP2 are thought to act as a factor critical for stabilization of these microdomains [49, 141]. Lamins itself can bind chromatin and DNA in vitro and in vivo [209, 256]. Recent direct evidences based on studies of HGPS patient cells demonstrated the involvement of progerin in abnormal chromatin anchorage, chromosome positioning and chromatin modification [152].

Silencing at the nuclear periphery is probably locus-specific and based on locus-specific mediator protein being able to interact transiently with NL proteins [121]. Tissue-specific genes that are co-expressed are located in clusters close to the nuclear periphery. Recent studies confirm that B-type lamins play an important role in silencing of multigenic regions and their transcriptional regulation. In *D. melanogaster* somatic cells, removal of lamin Dm results in release of the testis-specific cluster from the NE. Moreover, activation of the repressed gene cluster seems to be connected with its displacement from the NE [216]. This phenomenon is of increasing interest to scientists because it may allow them to explain the underlying mechanism of differentiation and development in normal and pathological conditions.

Studies of Hutchison–Gilford progeria syndrome (HGPS) and mutated lamin and proteins interacting with lamins revived and confirmed earlier studies regarding direct and indirect involvement of lamins and lamin containing complexes in chromatin binding and DNA-binding in vivo and its effect on chromatin organization and gene positioning and expression [9, 35, 40, 109, 118, 172, 173]. Lamins and protein complexes containing lamins can affect transcription via different mechanisms such as chromatin reorganization through recruitment of heterochromatin proteins, through recruitment of histone modifying protein complexes or transcription factors and silencers [261].

## Nucleus positioning

Nuclei as well as other organelles are non-randomly positioned in the cells. The location of the nucleus is precisely determined within the cell and its disruption may lead to serious dysfunction and decrease in cell viability [48, 140]. This may be exemplified by the phenomenon occurring in syncytial muscle cells in which the majority of nuclei are lined up separately beneath the plasma membrane whereas a small, distinctive population is clustered beneath the neuromuscular junction. Clustering is mediated by Syne1 protein responsible for positioning nuclei involved in synaptic activity due to the transcription of neuromuscular junction-specific genes (NMJ) in these nuclei [8]. Function of LINC complex proteins in nucleus positioning and neuromuscular junctions development has been described recently by many authors [39, 153, 221, 222, 247].

Many studies have led to the conclusion that there must be links (responsible for information exchange) between extracellular matrix constituents such as integrins and dystroglycan receptors and the cytoskeletal elements actin and intermediate filaments which in turn join the cytoplasmic environment (and thereby indirectly external milieu) with the nuclear interior [97], mainly via the outer nuclear membrane, inner nuclear membrane as well as NE components [232]. These also include nesprins, SUN domain proteins and lamins—all associated with the LINC complex. The importance of proper functioning of this multicomponent bridge, with reference to the muscle activity, has been well documented—mostly due to the detailed analysis of abnormalities caused by mutations in genes coding for its elements and resulting in muscular dystrophies or other myopathies {Ostlund, 2009 #5147).

It is known that myogenic transcription factors such as MyoD and myogenin can be induced by mechanical stimulation of cells (e.g. C2C12) and that this induction is strictly associated with LINC complex activity [33].

Disruption of the LINC complex in muscle cells affects their mechanotransduction properties [93]. This is reflected in, *inter alia*, changes of nuclear movements and muscle differentiation. In the case of C2C12 myoblasts, their differentiation also can be inhibited by aberration in the LINC complex. Abnormalities in this structure cause elevation of stretch-induced myogenic transcription factors (*inter alia*, by increase in p38 MAPK phosphorylation and subsequent augmentation of MyoD and myogenin levels) and inhibits stretch-induced proliferation). [33] (Fig. 3i).

Furthermore, mechanical stimulation of cells may result in deformations of the nucleus and thereby lead to genetic material rearrangements resulting in alteration of transcription factors’ access to the chromatin, thus affecting gene expression. This scenario seems to be plausible due to the existence of a microdomain in chromatin supported by the NE or NL proteins [49, 141, 180, 200, 217].

## Nuclear lamina and nuclear envelope proteins interactions with transcription factors

Lim-domain only 7 (Lmo7) is a transcription factor which activates expression of emerin and other heart and skeletal muscle genes. In mice, deletion of the gene coding for Lmo7 causes dystrophic phenotype. Both observations suggest that Lmo7 may be engaged in the EDMD mechanism by reduction of expression of emerin or other proteins important for muscle functioning [101]. Emerin binds to and inhibits the activity of Lmo7 [63]. Among genes controlled by Lmo7, four (*CREBBP*, *NAP1L1*, *LAP2*, *RBL2*) are misregulated in mice and patients lacking emerin. Lmo7 mediates transduction of mechanical or chemical signals between the nucleus and cytoplasm due to its association with the plasma membrane junctional complexes. This allows Lmo7 to induce the transcriptional response in cardiomyocytes. An analogical mechanism has been proposed in skeletal muscles, where transcription could be activated via changes in muscle attachment to the extracellular matrix [15, 101, 194]. Recent studies provide new data on the functional significance of Lmo7 and emerin interaction during myoblast differentiation [63]. According to the authors, Lmo7 is necessary for proper C2C12 myoblast differentiation due to its ability to activate *Pax3*, *MyoD* and *Myf5* expression by binding to their promoters. Interactions between Lmo7 and these promoters are facilitated by its nucleoplasmic localization. As the differentiation proceeds, probably via a change in the nuclear transport dynamics, Lmo7 localizes mainly in the cytoplasm of maturing myotubes. Affinity of emerin to Lmo7 increases, leading to inhibition of Lmo7 interactions with promoters of genes engaged in muscle differentiation [63] (Fig. 3b).

Emerin interacts with germ cell-less (GCL), a transcription repressor which inhibits E2F-DP dependent gene expression by direct binding to the subunit of E2F-DP dimer [100, 101, 244]. Another emerin partner, transcription factor Btf, may be linked to the EDMD disease because two mutations in emerin, S54F and Δ95-99, disrupt their interaction [94]. Emerin can be regulated by phosphorylation because 13 of its 18 tyrosine residues are phosphorylated in vivo. Recently three tyrosine kinases, Her2, Scr and Abl, that control emerin modification were identified [230]. Human epidermal growth factor receptor 2 (Her2, encoded by the *ERBB2* gene) belongs to the epidermal growth factor receptor family. This surface membrane-bound protein is involved in the signal transduction pathways responsible for tissue growth and differentiation, including heart and skeletal muscles [176]. Scr and Abl are probably downstream effectors of Her2 directly phosphorylating human emerin [206]. In diseased heart and skeletal muscles (emerin-deficient), the Her2 signaling pathway is disrupted. These findings may suggest that emerin plays a role as a downstream effector of Her2 and is a potential integrator of signals from different pathways [139, 230]. Another LEM domain protein, LAP2β, is also phosphorylated by Scr and Abl [230]. Moreover, Her2-mediated phosphorylation regulates association of emerin with BAF. It is worth noting that under normal conditions, skeletal muscle nuclei located beneath the neuromuscular junction are under the control of neuregulin-Her2 signaling, which regulates expression of genes required for neuromuscular junction integrity. However, Mejat et al. [154] demonstrated that the neuromuscular junctions in AD-EDMD patients and a mouse model of the disease (H222P) are defective. Mutation of lamin A, which also takes part in the positioning of the synaptic nuclei at the neuromuscular junction in muscles, prevents it from performing its function. Nuclei are mislocalized at the neuromuscular junction and NMJ-specific genes are misregulated in both mice and humans [154].

Tifft et al. [230] based on their own and previous findings, proposed the “disrupted signaling” hypothesis for EDMD diseases, according to which emerin functions as a signal integrator at the NE taking part in tyrosine kinase signaling by providing the “scaffold” for Scr targets. These conclusions were drawn from the observations indicating that emerin plays a crucial role in the regulation of β-catenin and Lmo7 activity, and its loss or lack of phosphorylation influences JNK, MAPK, integrin, Wnt, TGFβ and fibroblast growth factor receptor 2IIIb (FGFR2IIIb) signaling pathways [101, 144, 166, 230].

## The involvement of other nuclear membrane proteins in the pathogenesis of EDMD

Besides mutations in genes coding for emerin and lamin A, also changes in genes encoding nesprin-1 (*SYNE1*) [194, 195] and nesprin-2 (*SYNE2*) [259] lead to EDMD. The fact that these four proteins interact with each other suggests a common disease mechanism based on disruption of the nucleo-cytoskeletal junction [12, 21, 32, 122, 242]. In mice, deletion of genes encoding nesprin-1 and -2 or overexpression of the KASH domain, which destroys endogenous LINC complexes, results in, besides the disruption of nuclear organization, failure of nuclei to cluster at the neuromuscular junctions [86, 260].

In order to explain the mechanism of EDMD resulting in nesprin mutations, mice with truncated nesprin-1 lacking the C-terminal KASH domain have been created [194]. Approximately 50 % of homozygous mice exhibit perinatal lethality, whereas survivors indicate progressive muscle weakness (particularly hind limb), gait disturbance and cardiac defects. All double mutant mice (Syne-1 and Syne-2 null) died perinatally or immediately after birth due to the respiratory failure [260] indicating that nesprin-1 and nesprin-2 may partially overlap their structural function and positioning of nuclei at the neuromuscular junction. Indeed, during the maturation of myotubes, nesprin-1 is gradually replaced by nesprin-2 [199]. In cardiac and skeletal muscles from EDMD patients, as well as in the mutant mice, components of the LINC complex are not mislocalized (including truncated nesprin-1α and its binding partner SUN2). However, the LINC complex displays abnormalities in association between mutant nesprin-1 and SUN2. Thus loss of the bridge between the nucleoplasm and cytoplasm may be the cause of neuromuscular diseases [194]. Moreover, emerin and short nesprin-2α isoform (which lacks the KASH domain and is located at the INM via its associations with lamin A and emerin) are identified as novel binding’s partners of SUN1 and SUN2 [259]. Interactions of both SUN proteins are perturbed in EDMD and HGPS. Since SUN1 and SUN2 play critical, but overlapping functions in anchoring nuclei in skeletal muscles [127] this may suggest that SUNs also participate in the development of these diseases [93].

## Prospects for treatment

Over the last decade, several reports have been published on the possible use of small molecules for laminopathy treatment. Molecular targets for treatment are usually key enzymes from signaling or metabolic pathways giving rise, with combination with mutated gene, to specific laminopathy. For lipodystrophy, typically leptin or thiazolinidone (TZD) have been recommended. For HGPS treatment, the combination of N-containing bisphosphonates, statins and farnezyl transferaze inhibitors (FTIs) (e.g. tipifarnib, lonafarnib) have been used in clinical trials ([81, 237, 249] and Progeria Research Foundation webpage). Unfortunately both statins and FTIs are likely to cause significant side effects. FTIs are also modulators of activity of membrane-bound small GTPases and reportedly induce “donut-shaped cell nuclei”—the effect associated with centrosomes separation defect [238].

Another small molecule: *N*-acetylcysteine can be used to prevent the oxidative damage of DNA in progeria HGPS and other laminopathies since it has been positively tested in mdx mice model [229].

Recently, rapamycin was positively tested on HGPS fibroblasts in removal of nuclear blebbing and delaying cellular senescence [40]. This discovery is especially interesting because rapamycin affects mTOR signaling pathway and, among others, modulate IGF-II signaling. Recently Carlos-Lopez-Otin group introduced recombinant IGF-1 as a useful treatment for HGPS [147]. Another possible treatment for laminopathies suggested the use of histone deacetylase inhibitors (e.g. trichostatin A) [54] and inhibitors of mitogen activated protein kinases (ERK1-2) [170] and in general inhibitors of signaling tyrosine kinases for “muscular” laminopathies. Recently, induced macrophagy was suggested as possible treatment for HGPS [44, 87]. Unfortunately, all current pharmacological approaches to laminopathies did not give satisfactory results and are not targeted to cure but only to ameliorate the effects and slow down or stop the disease progression. Perhaps, the advances made in gene therapy development for other muscular dystrophies such as Duchene and Becker muscular dystrophy (DMD and BMD) may provide the solution. For example, *N*-Acetylcysteine may be used to prevent the oxidative damage of DNA in progeria HGPS and other laminopathies since it has been positively tested in mdx mice model [229].

Transdifferentiation as an alternative method for generation of muscle cells for patient treatment and research has also been considered. Several studies have demonstrated that ectopic expression of MyoD acts as a reprogramming factor and is sufficient for the transdifferentiation of non-muscle cells such as dermal fibroblasts, chondroblasts, gizzard smooth muscle, and pigmented retinal epithelial cells into myoblasts [19]. In addition, ectopic expression of Pax7 seems to be sufficient to reprogram ES and iPS cells to muscle progenitor cells [59, 60]. Exogenous MyoD enables muscle formation which is indiscernible from normal myogenesis with preservation of steps occurring in vivo and in vitro, including cell cycle withdrawal, subsequent expression of the proteins involved in the myogenic program (desmin, α-actinin, titin, troponin I, α-actin, myosin heavy chain) and fusion of the myoblasts into multinucleated myotubes (independent of surrounding extracellular matrix molecules). Activation of myogenesis leads to down regulation of the original differentiation program of the converted cell [64]. Transdifferentiation of various types of cell has been considered as medical treatment. Application of easy-to-obtain and abundant cells, especially fibroblasts, as potential therapeutic agents appears to be an interesting variant in myogenic [106] and cardiomyogenic diseases [228]. Further investigation of this absorbing phenomenon may provide us an invaluable tool to replace damaged tissue.

Recently, reproducible procedures for transdifferentiation have been established starting from somatic cells from patients with different diseases. Reprogrammed cells, called induced pluripotent stem cells (iPSCs), also displayed a particular disease phenotype and thus can be used as a model for differentiation studies. From the point of view of laminopathies, two iPSC models have been established recently: for autosomal dominant Emery–Dreifuss muscular dystrophy (AD-EDMD) [98] and for Hutchinson–Gilford progeria syndrome (HGPS) [258]. The major disadvantage of ES and iPS cells is their potential for cancerogenesis [113].

Adult mesenchymal stem cells (MSCs) that can be isolated from bone marrow appear to be a useful source of material for tissue transplantation techniques and regenerative medicine since they can differentiate into various tissue types including muscles. The MSCs have a large capacity for self-renewal and in tissue cultures are able to maintain their multipotency. Another advantage of the MSCs is their capacity of secretion of a broad spectrum of bioactive molecules that ensure a regenerative environment for various tissues. Some investigations suggest that the MSCs are a suitable tool for treatment of myocardial infarcts [42]. Although some reports indicate that myoblasts and nuclei from myotubes originated from HSC cells may not be fully functional with respect to expression of muscle-specific proteins [50], they may have beneficial effect by creating stem cell environment and induce effective regeneration of diseased muscles [90]. It has also been reported that apart from fibroblasts, human synovial stem cells (hSSCs) could be considered as an appropriate agent for clinical applications. These cells display the ability to regenerate muscle fibers and the reservoir of satellite cells, features that can be enhanced by ectopic expression of human MyoD [157]. Although, efficiency of the muscle regeneration process of hSSCs is not satisfactory, their capacity for expression and secretion of extracellular matrix components (including laminin α2 and collagen VI) makes them a useful tool in the search for treatments to help fight muscular dystrophies [189]. Intra-muscular transplantation of cells that are able to play a trophic role may prove to be helpful during the development of novel therapies for muscle diseases caused by deficiencies in their microenvironment [190].

It is hard to imagine at this moment what would be the future cell and gene therapy for laminopathies. Researchers still suffer from insufficient knowledge of the molecular background of the diseases. Nevertheless, attempts at such treatment should be made at least on tissue-cultured patient cells and model organisms. Such attempts can be based on the current knowledge about cell and gene therapy for other muscular dystrophies. Especially, Duchene muscular dystrophy (DMD) or limb-girdle muscular dystrophy 2E/2F (LGMD) and experiments on a mice model system for these diseases (mdx and sgcb-null mice respectively) seem to be the most optimistic [90, 91]. The strategy for treatment of autosomal recessive types of laminopathies looks pretty straightforward; it is only necessary to deliver a wild type copy of the defective gene into the majority of the myotubes and provide the proper expression cassette in order to obtain the desired level of wild type protein expression. Autosomal dominant diseases, when the mutated protein has the property of “negative dominant mutant”, however, require a much more complex approach.

Generally, it requires the neutralization of the mutant allele (protein). This can be done either by the lentivirus mediated si/shRNA method or homologous recombination of the mutated genomic sequence with a specially prepared vector silencing the gene. This strategy may have to be simultaneously accompanied by insertion of a wild type allele into the genomic DNA. The si/shRNA method and homologous recombination/gene editing methods have been successfully used in HGPS treatment in a tissue culture model [102, 133, 211]. Recently, induced macrophagy was suggested as possible treatment for HGPS [44, 87].

Unfortunately, our limited knowledge of the genetic manipulation of muscle cells in mice and humans renders gene editing and the homologous recombination approach of gene therapy technically impossible to apply, mostly due to the limited efficiency of such procedures in vivo. There are many different reasons why this is the case. Some of them originate in the properties of muscles and their compact structure, the presence of the basal lamina limiting access to each myotube as well as heterogeneity of cells and nuclei inside the basal lamina. Other technical limitations lie in difficulty in targeting vectors onto myotubes and poor efficiency of transfection or transduction of myotubes. Another set of problems arises from the time span in which the therapeutic construct is maintained in myotubes: whether the construct is integrated with genomic DNA, whether the protein is expressed in sufficient amounts and whether myotubes expressing the wild type protein are eliminated by the immune system. Another important technical difficulty is presented when gene therapy is undertaken on adult subjects. The restoration of the original mass and efficiency of muscles when muscles have already been severely damaged by the disease poses a problem. Specifically, the problem is how to induce genetically modified satellite cells or side population cells to proliferate, migrate and restore wasted muscle fibers.

There should be no such problem in young organisms, at least in the case of mice. There is evidence from mice models that the earlier gene therapy is intiated, the better and the more profound are the effects that can be achieved. This is probably due to the ability to modify a larger population of satellite cells and newly forming myotubes in young organisms. Young (developing) muscle tissue may also be more permeable than adult tissues.

Some published data [156] obtained on mdx and sgcb-null mice transduced with virus vectors carrying dystrophin and sarcoglycan genes respectively are very promising. Unfortunately, the mouse model system for dystrophies is not the best for developing gene therapy for human dystrophies, mostly due to the vastly different lifespan of mice and humans and in consequence insufficiently long maintenance of the therapeutic vector or persistence of modified cells. The above-mentioned problems encountered by systemic gene therapy for muscular dystrophies are only a few examples of the most obvious among many others of varying importance.

Fortunately there are other, theoretically possible strategies for cell and gene therapy treatment for muscular dystrophies. They are mostly based on ex vivo gene therapy based on the use of muscle satellite cells (or side population cells), pluripotent mesenchymal stem cells (MSCs) or hematopoietic stem cells (HSCs), synovial cells or the use of terminally committed cells such as fibroblasts reprogrammed by transdifferentiation into MSCs or myoblasts [208]. Such strategies although promising, also face several technical difficulties. It is generally known how to isolate MSCs or HSCs (CD133+) and differentiate them into myoblasts in vitro. There is essentially no technical problem with transfection and transduction of such cells or with selection and propagation. It is also possible to transdifferentiation fibroblasts into myoblasts.

The main problems with the ex vivo strategy are associated with problems in keeping the stemness of cells and (especially muscle stem cells) and accessibility of muscles to such prepared cells. A series of intramuscular microinjections is only possible in the case of easily accessible muscles. Systemic delivery into the blood system requires the use of an efficient targeting system enabling such cells to be retained efficiently in muscle blood vessels followed by migration into the muscle tissues. At this point the question arises which ex vivo modified cells should be used: myoblasts, HSCs, MSCs or perhaps synovial cells [3, 4]?

Myoblasts have a significant advantage over HSCs when direct microinjection into mice heart is analyzed due to the better migration potential. When systemic delivery is considered, HSCs appear to have an advantage over any other cells. Considering all possible strategies of ex vivo gene therapy for muscular dystrophies, HSCs and MSCs display a significant advantage over other cells because they can differentiate into myoblasts and subsequently into myotubes, may be easily delivered to damaged tissue or can be stimulated to differentiate in vitro and then delivered to patients. Thus, efficient gene therapy for muscular laminopathy may be based on the isolation of HSCs from the potential patient, correction of the disease phenotype and co-transfection with the genes encoding proteins responsible for differentiation into “primary” myoblasts such as Shh, Pax7, MyoD, and Sca-1. Obtained cells would be infused or microinjected back into patients, where they should differentiate into primary myoblasts and then into muscle cells, which can repopulate and fuse with existing myotubes or form new muscles.

## Conclusions

Although laminopathies are relatively rare diseases comparing to Duchenne or Becker muscular dystrophy (DMD), investigations of these abnormalities may bring valuable information about general processes occurring in cells under normal (e.g. physiological ageing) as well as pathological conditions such as muscular dystrophies.

A better understanding of mechanisms leading to diseases may contribute to discovery of novel therapies. Recent studies suggest some solutions that could be used as medical treatment. For example, improvement of differentiation in C2C12 myoblasts expressing R453W mutant lamin A is possible after pharmacological inhibition of the MAPK pathway [71]. An earlier study proposed another way of increasing the differentiation potential of lamin A-null myoblasts by providing them with exogenously expressed desmin or MyoD [75].

Also, development of an application using transdifferentiation of donor engineered cells may prove to be an invaluable tool for treatment of various dystrophic diseases.

Experiments undertaken over the last 10 years have shown that we are at the beginning of understanding the role that NE proteins play in the complex network regulating cell function. Comprehension of all molecular connections is a great challenge. However, the tangled web of mutual relations is an obstacle hindering explanation of the mechanisms underlying the development of laminopathies.
